# Genome-Wide Association Study for Milk Fatty Acids in Holstein Cattle Accounting for the *DGAT1* Gene Effect

**DOI:** 10.3390/ani9110997

**Published:** 2019-11-19

**Authors:** Valdecy A. R. Cruz, Hinayah R. Oliveira, Luiz F. Brito, Allison Fleming, Steven Larmer, Filippo Miglior, Flavio S. Schenkel

**Affiliations:** 1 Centre for Genetic Improvement of Livestock, Department of Animal Biosciences, University of Guelph, Guelph, Ontario, ON N1G 2W1, Canada; valdecya.r.cruz@gmail.com (V.A.R.C.); hinayah@gmail.com (H.R.O.); britol@purdue.edu (L.F.B.); afleming@lactanet.ca (A.F.); slarmer@semex.com (S.L.); fmiglior@ontariogenomics.ca (F.M.); 2 Department of Animal Sciences, Purdue University, West Lafayette, IN 47907, USA; 3 Lactanet Canada, Guelph, Ontario, ON N1K 1E5, Canada; 4 Ontario Genomics, Toronto, Ontario, ON M5G 1M1, Canada

**Keywords:** *DGAT1*, *MGST1*, milk fat composition, milk properties, North American Holstein cattle, *PLBD1*, saturated fatty acid, unsaturated fatty acid

## Abstract

**Simple Summary:**

Milk fat content and fatty acid composition are key traits for the dairy industry, as they directly influence consumer acceptance of dairy products and are associated with the chemical-physical characteristics of milk. In order to genetically improve milk fat composition, it is important to understand the biological mechanisms behind the phenotypic variability observed in these traits. In this study, we used a genomic dataset for 6692 animals and over 770,000 genetic markers distributed across the genome. We compared different statistical approaches to better identify the genes associated with milk fatty acid composition in Holstein cattle. Our results suggest that the *DGAT1* gene accounts for most of the variability in milk fatty acid composition, and that the *PLBD1* and *MGST1* genes are important additional candidate genes in Holstein cattle.

**Abstract:**

The identification of genomic regions and candidate genes associated with milk fatty acids contributes to better understand the underlying biology of these traits and enables breeders to modify milk fat composition through genetic selection. The main objectives of this study were: (1) to perform genome-wide association analyses for five groups of milk fatty acids in Holstein cattle using a high-density (777K) SNP panel; and (2) to compare the results of GWAS accounting (or not) for the *DGAT1* gene effect as a covariate in the statistical model. The five groups of milk fatty acids analyzed were: (1) saturated (SFA); (2) unsaturated (UFA); (3) short-chain (SCFA); (4) medium-chain (MCFA); and (5) long-chain (LCFA) fatty acids. When *DGAT1* was not fitted as a covariate in the model, significant SNPs and candidate genes were identified on BTA5, BTA6, BTA14, BTA16, and BTA19. When fitting the *DGAT1* gene in the model, only the *MGST1* and *PLBD1* genes were identified. Thus, this study suggests that the *DGAT1* gene accounts for most of the variability in milk fatty acid composition and the *PLBD1* and *MGST1* genes are important additional candidate genes in Holstein cattle.

## 1. Introduction

Milk fat composition is directly related to nutritional aspects of milk and dairy products, which account for up to 30% of the total fat intake in the human diet [1]. Several studies have documented the potential effects of dietary fatty acids on human nutrition, as well as the recommended limit of dietary fat intake [2,3,4]. Milk fat is composed of a complex mixture of lipids, which contains approximately 70% saturated (SFA) and 30% unsaturated (UFA) fatty acids [5,6]. Some fatty acids are essential nutrients for human health, such as phospholipids, that are important constituents of cell membranes [7]. Nonetheless, the high SFA content in milk is often undesirable, as it has been associated with several diseases, including elevated blood cholesterol, obesity, and cardiovascular problems [2,8,9].

Saturated fatty acids and UFA of different chain lengths have ranging heritability estimates. For instance, short- (SCFA) and medium-chain (MCFA) fatty acids have shown moderate to high heritability estimates, while long-chain fatty acids (LCFA) are usually low to moderately heritable [10,11,12]. In addition to genetic parameters, a better understanding of the milk fatty acid synthesis through the identification of important genomic regions and candidate genes can contribute to faster genetic progress for such traits. This can be done by incorporating this information into the genomic prediction of breeding values through methods that account for differential influences of specific genomic regions.

Genome-wide association analysis (GWAS) is the gold-standard method by which to identify genetic variants and genomic regions that are significantly associated with complex traits. It then contributes to the understanding of the genetic architecture and underlying biology of milk fatty acid synthesis. In GWAS, thousands of single nucleotide polymorphisms (SNPs) are tested for association with the trait(s) of interest by exploiting the linkage disequilibrium that exists between the causative mutation and one or more genetic markers [13]. Recent studies have reported that genes located on BTA5, BTA14, and BTA20 are significantly associated with milk fatty acids [8,13,14]. However, except for major genes such as the *Diacylglycerol O-acyltransferase 1* (*DGAT1*), there is a limited consensus on which genes are involved in milk fatty acid composition. This may be related to the fact that major genes can be in linkage disequilibrium with several SNPs, and thus, can hinder the identification of additional genes with minor effects [15]. In this context, fitting the effect of major genes in the statistical model could be an alternative approach to identify additional candidate genes with smaller effects on the trait(s) of interest [16]. Therefore, the objectives of this study were: (1) to perform GWAS for five groups of milk fatty acids in a North American Holstein population using a high-density SNP panel; and (2) to compare the results of GWAS when fitting (or not) the *DGAT1* gene effect as a covariate in the GWAS models.

## 2. Materials and Methods

### 2.1. Datasets

Five groups of predicted milk fatty acids were analyzed in this study: (1) saturated fatty acids (SFA); (2) unsaturated fatty acids (UFA); (3) short-chain fatty acids (SCFA), which include fatty acids with 4 to 10 Carbons; (4) medium-chain fatty acids (MCFA), which include fatty acids with 12 to 16 Carbons; and (5) long-chain fatty acids (LCFA), which include fatty acids with 17 to 22 Carbons. The fatty acid composition, in g/dL of milk, were predicted based on milk mid-infrared spectroscopy. Details about the prediction equations and phenotypic quality control are fully described in Fleming et al. [17] and Narayana et al. [11]. For all groups of milk fatty acids, 52,592 records from the first lactation of 14,129 Holstein cows were available. A total of 215,233 animals were included in the pedigree.

A total of 3121 animals were genotyped using low- or medium-density SNP panels (7K, 8K, 15K, 20K, or 30K), 3676 animals using a 50K SNP panel, and 1115 animals using a high-density (HD; 777K) SNP panel. Animals genotyped with the low- or medium-density panels were initially imputed to the 50K panel and, thereafter, all 50K genotypes were imputed to 777K. Genotype imputation was performed using the FImpute software (Guelph, ON, Canada) [18]. Imputation accuracy from 50K to 777K in this population is greater than 0.95, as reported in Larmer et al. [19]. After the genotype imputation process, SNPs with a call rate less than 0.95, a minor allele frequency (MAF) less than 0.01, significant deviation of the expected Hardy-Weinberg equilibrium (*p* < 10^−6^), and SNPs in high linkage disequilibrium (r^2^ > 0.90) were removed. A total of 6692 genotyped animals and 610,272 SNPs distributed across the 29 bovine autosomes (BTA) remained for further analyses.

### 2.2. Variance Components, Breeding Value Estimation and Deregression Analyses

Preliminary analyses (including descriptive statistics, normality test, and test of fixed effects to be included in the statistical model) were performed using the UNIVARIATE and GLM procedures in the Statistical Analysis System (SAS) software (version 9.2, SAS Institute, Inc. 2011, Cary, NC, USA). Variance components and estimated breeding values (EBVs) were calculated using the ASReml v3.0 software (Hemel Hempstead, UK) [20]. The statistical model used can be described as follows:

**y** = **Xb** + **Za** + **Wpe** + **Qhy** + **e** (1)

where **y** is the vector of phenotypic records, **b** is the vector of fixed effects, **a** is the vector of random animal additive genetic effects, **pe** is a vector of random permanent environment effects, **hy** is a vector of random herd-year effects, and **e** is a vector of random residual effects. **X**, **Z**, **W**, and **Q** are the corresponding incidence matrices. The significant (*p* < 0.05) fixed effects that were included in the final statistical model are herd-test day, season of calving, and days in milk (DIM; split into 12 equidistant classes). It was assumed that E(**y**) = **Xb**, **a** ~ N(0, **A**$\sigma_{a}^{2}$), **pe** ~ N(0, **I**$\sigma_{p}^{2}$), **hy** ~ N(0, **I**$\sigma_{\mathrm{hy}}^{2}$), and **e** ~ N(0, **I**$\sigma_{e}^{2}$), where **A** is the pedigree-based relationship matrix, **I** is an identity matrix, and $\sigma_{a}^{2}$, $\sigma_{p}^{2}$, $\sigma_{\mathrm{hy}}^{2}$, and $\sigma_{e}^{2}$ are the additive genetic, permanent environment, herd-year, and residual variances, respectively.

Estimated breeding values were deregressed using the VanRaden and Wiggans method [21] in order to avoid the double-counting of information and to increase the variability of EBVs [22]. Thereafter, deregressed EBVs were used as pseudophenotypes in the GWAS. Pseudophenotypes were used, as not all phenotyped animals were genotyped.

### 2.3. Genome-Wide Association Analyses

The GWAS were carried out based on single-SNP regression, using the allele substitution model implemented in the SNP1101 software [23]. In order to contrast the results of GWAS fitting (or not) the *DGAT1* gene as covariate in the GWAS model, two approaches were compared for each milk fatty acid trait. The first model is described as follows:

**y** = **1**μ + **S**m + **Zu** + **e** (2)

where **y** is the vector of deregressed EBVs, µ is the population mean, m is the fixed effect of the SNP genotype, **u** is the vector of random genomic effects (in order to account for the polygenic effect), **1** is a vector of ones, **S** and **Z** are the incidence matrices related to **m** and **u**, respectively, and **e** is the vector of residuals. It was assumed that **u** ~ N(0, **G**$\sigma_{u}^{2}$), where **G** is the genomic relationship matrix, and $\sigma_{u}^{2}$ is the genomic variance (i.e., the genetic variance estimated based on the **G** matrix). The same **G** matrix was used in all analyses performed in this study (i.e., markers located in the genomic regions under examination were not removed when constructing the **G** matrix), due to the small impact that it would have in the GWAS analyses [24]. Therefore, **G** was created as proposed by [25]:

(3) $$G=0.95\frac{{\mathrm{WW}}^{\prime }}{2\sum_{K=1}^{K}p_{K}\left(1-p_{K}\right)}+0.05A$$

where **W** = **M** − **P**, in which **M** contains the centered genotypes (i.e., −1, 0 and 1 to represent AA, AB and BB, respectively), **P** contains the allele frequency for SNP k (p_k_) in its k^th^ column, expressed as 2(p_K_ − 0.5), and $2\sum_{K=1}^{K}p_{K}\left(1-p_{K}\right)$ is a scaling parameter.

The second GWAS model considered the SNP with the highest allele substitution effect in the *DGAT1* gene region as a fixed effect. The top SNP was determined based on the first model. The *DGAT1* gene was previously reported as the major gene for milk fat yield and milk fatty acids in Italian Simmental and Holstein [8]. Therefore, the top SNP was fitted as a covariate in the second model as follows:

**y** = **1**μ + DGAT1 + **S**m + **Zu** + **e** (4)

where DGAT1 is the fixed effect that accounts for the allele substitution effect of the SNP related to the *DGAT1* gene. All the other terms in the model were previously defined above. In order to account for multiple testing, a genome-wide false discovered rate (FDR) was applied to indicate significant association between SNP and each fatty acid group. The SNPs were considered for further analyses when the *q-value* of the genome-wise FDR was lower than 1, 5, or 10%. The markers above the 5% threshold were interpreted as suggestive SNPs.

### 2.4. Candidate Genes, Enrichment and Biology Pathway Analysis

Significant SNPs were used to identify candidate genes, using the NGS-SNP collection of command-line scripts [26]. Thereafter, gene networks and biological pathways were identified using the KEGG (www.genome.jp/kegg/genes.html) and Gene Cards (https://www.genecards.org) tools.

## 3. Results

### 3.1. SNP Variances and Number of Significant SNPs

The numbers of significant SNPs for the different groups of milk fatty acids, either fitting or not the *DGAT1* gene in the statistical model, are shown in Table 1. A higher number of SNPs was significant when the *DGAT1* gene was not fitted independently in the model, for all groups of milk fatty acids. When fitting the *DGAT1* gene as a covariate in the model, only a few significant SNPs were identified, especially for MCFA and SCFA. A ranking of SNP variances when not fitting (model 1) or fitting (model 2) the *DGAT1* gene as a covariate in the statistical model is presented in the Supplementary File 1. In general, large differences in rank were observed between the two models, and larger variances for the significant SNPs were estimated by model 1 compared to model 2.

### 3.2. Genomic Regions and Candidate Genes

**Figure 1:**
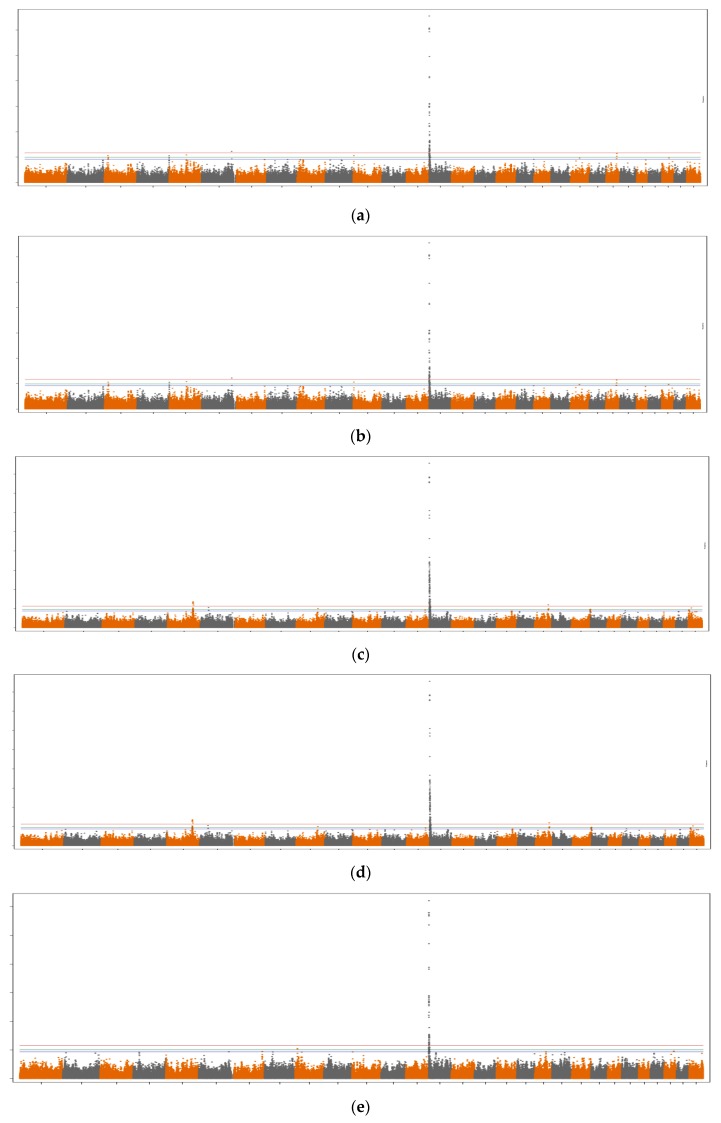
Manhattan plots for the different fatty acid groups [i.e., long-chain (**a**), medium-chain (**b**), short-chain (**c**), saturated (**d**), and unsaturated (**e**) fatty acids], when the *DGAT1* gene was not fitted as a covariate in the statistical model. Genome-wide plots have the -log10 (I-values) in the y-axis and the chromosome number in the x-axis. Lines represent chromosome-wise FDR thresholds of 10% (blue), 5% (green), and 1% (red).

**Figure 2:**
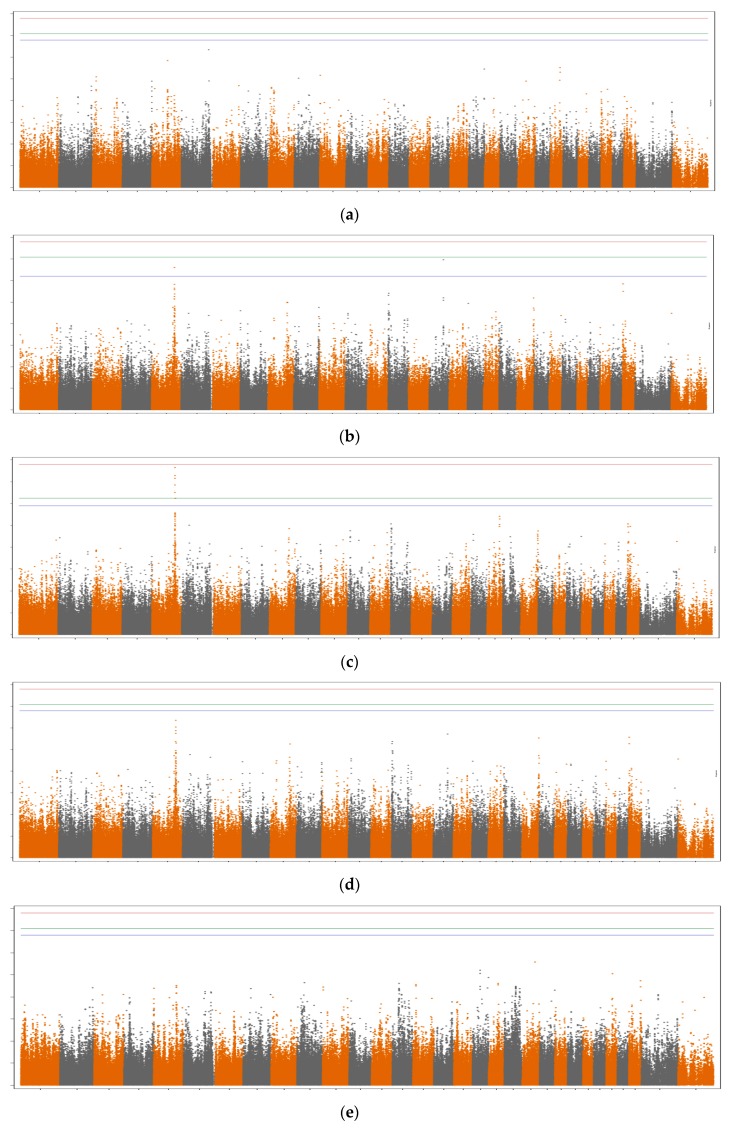
Manhattan plots for the different fatty acid groups (i.e., long-chain (**a**), medium-chain (**b**), short-chain (**c**), saturated (**d**), and unsaturated (**e**) fatty acids), when fitting the *DGAT1* gene as a covariate in the statistical model. Genome-wide plots show the -log10 (I-values) in the y-axis and the chromosome number in the x-axis. Lines represent chromosome-wise FDR thresholds of 10% (blue), 5% (green), and 1% (red).

Manhattan plots for the different groups of fatty acids are shown in Figure 1 and Figure 2, when not fitting and fitting the *DGAT1* gene as a covariate in the GWAS model, respectively. A similar pattern was observed between the different Manhattan plots, even though the majority of SNPs were not significant when *DGAT1* was fitted as a covariate in the model (Figure 2).

When not fitting the *DGAT1* gene as a fixed effect in the model, significant SNPs were found on BTA5, BTA6, BTA14, BTA16, and BTA19. This is likely due to the fact that the candidate genes located in these regions are involved in similar pathways as the *DGAT1* gene. A high proportion of overlapping regions was observed among the different groups of fatty acids. For instance, an important region on BTA14, which includes the *DGAT1*, *FOXH1,* and *CYHR1* genes, was associated with all fatty acid groups. Moreover, an important region was detected on BTA5 for the majority of fatty acid groups, which includes the *MGST1* and *PLBD1* genes. In addition, not fitting the *DGAT1* gene as a covariate in the statistical model allowed us to identify new, significant regions on BTA16 (unknown protein) and BTA19 (including the *LOC101909618* gene). However, when the *DGAT1* gene was fitted in the model, significant genomic regions were found only on BTA5 and BTA16 for specific groups of milk fatty acids.

LCFA: As shown in Table 1 and Figure 1a, several significant SNPs were found for LCFA when the *DGAT1* gene effect was not fitted in the statistical model. In summary, significant SNPs were located on BTA14, close to the *DGAT1*, *FOXH1*, and *CYHR1* genes. In addition, a small significant region on BTA6 was found around position 111,458,055 bp, but no annotated genes were retrieved. No significant SNPs were found for LCFA when fitting the *DGAT1* gene in the statistical model (Table 1 and Figure 2a).

MCFA: Several significant SNPs were found for MCFA when the *DGAT1* gene was not fitted in the statistical model (Table 1 and Figure 1b). The significant SNPs were located on BTA14 (close to the *DGAT1*, *FOXH1*, and *CYHR1* genes), BTA5 (close to the *MGST1* gene), and BTA16 (unknown protein). SNPs located in similar regions on BTA5 and BTA16 were significant or suggestive (FDR=10%) when fitting the *DGAT1* gene effect as a covariate in the model (Table 1 and Figure 2b).

SCFA: When not fitting the DGAT1 gene in the model, several SNPs located on BTA14 were significant (Table 1 and Figure 1c). These SNPs were located in a region similar to that found for LCFA and MCFA, and were within or close to the *DGAT1*, *FOXH1*, and *CYHR1* genes. In addition, five SNPs on BTA5 were significant, which were within or harbored the MGST1 and PLBD1 genes. One significant SNP was also identified on BTA19, within the *LOC101909618* gene, when not fitting the *DGAT1* gene in the model. When fitting the *DGAT1* gene in the model, four SNPs on BTA5 were significant (FDR = 5%), within or close to the *MGST1* gene (Table 1 and Figure 2c).

SFA: Similar to LCFA, SCFA, and MCFA, several SNPs located on BTA14 were significant (Table 1 and Figure 1d) when the *DGAT1* gene was not fitted as a covariate in the statistical model. In addition, three significant SNPs were located on BTA5 (within or close to the *MGST1* gene), and one significant SNP was located on BTA16, around the region 93,945,655 bp (unknown protein). No significant SNPs were found for SFA when fitting the *DGAT1* gene in the model (Table 1 and Figure 2d).

UFA: A lower number of significant SNPs distributed across a smaller region was found for UFA when compared to the other groups of milk fatty acids. These significant SNPs were located on BTA14, in a region close to the *DGAT1*, *FOXH1*, and *CYHR1* genes (Table 1 and Figure 1e) when the *DGAT1* gene effect was not fitted as a covariate in the model. No significant SNPs were found for UFA when fitting the *DGAT1* gene in the model (Table 1 and Figure 2e).

### 3.3. Candidate Genes and Functional Analyses

**Figure 3:**
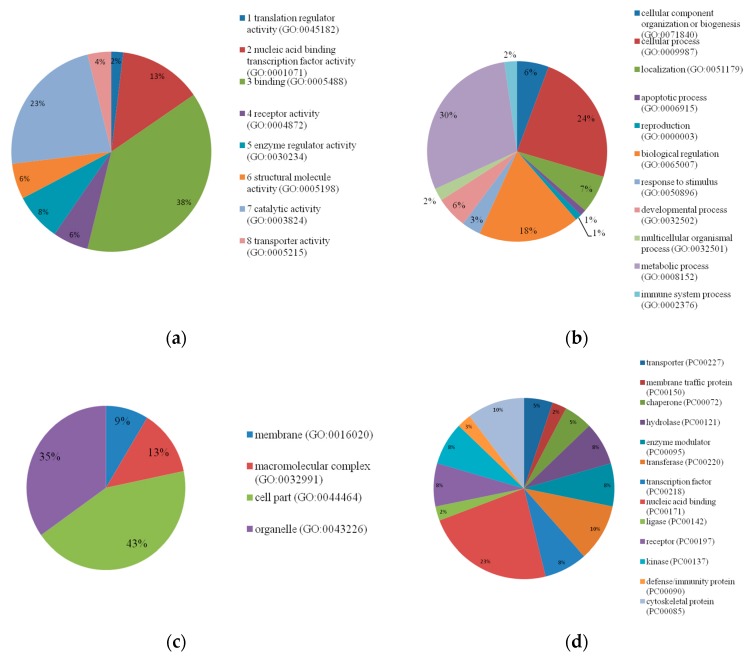
Functional analyses for the candidate genes found to be associated with milk fatty acids. Candidate genes were clustered based on four different potential expression profiles: molecular function (**a**), biological process (**b**), cellular component (**c**), and protein class (**d**).

Figure 3 shows the functional analyses for the candidate genes. In summary, the candidate genes were clustered into four different functional categories: molecular function, biological process, cellular component, and protein class. The categorization related to molecular function showed that the majority of profiles (38%) are involved in cellular binding. With regards to the activities related to biological process, three clusters were more evident: biological regulation (18%), cellular process (24%), and metabolic process (30%). For the cellular component, two main clusters were detected: organelle (35%) and membrane component (43%). The functional profiles related to protein class resulted in several small clusters. The largest one was involved in nucleic acid binding (23%).

## 4. Discussion

Genome-wide association analyses were performed for five groups of milk fatty acids in Holstein cattle. The majority of candidate genes identified are located on BTA14, which is in agreement with Li et al. [27] and Palombo et al. [8], who studied milk fatty acids in Chinese and Italian Holstein cattle, respectively. Most of the significant genomic regions found in this study are located on BTA14 within or harboring the *DGAT1*, *FOXH1,* and *CYHR1* genes. The *DGAT1* gene is well known due to its strong association with milk production traits, especially milk fat [27,28,29]. The *CYP11B1* gene, which is a *CYHR1* paralogous [30], has been reported to be associated with milk fat in buffalo [31]. Also, Boleckova et al. [32] reported that *CYP11B1* is a functional gene for milk production traits in cattle. However, the authors suggested that the effect of this gene may be affected by a strong linkage disequilibrium with polymorphisms in the *DGAT1* gene, which has been well described in the literature [33].

Even though the *FOXH1* gene is involved in lipid metabolism, *FOXO1* (*FOX family* gene) is an insulin-sensitive DNA binding transcription factor that can alter the synthesis or stability of the MAF1 protein. The *MAF1* gene (*MAF1* homolog, negative regulator of RNA polymerase III) is a protein coding that is important to the regulation of mammalian intracellular lipids [34,35]. The MAF family member proteins regulate tissue-specific gene expression and cell differentiation in a wide variety of tissues. For instance, it has been shown that the *MAFA* gene (*V-maf avian musculoaponeurotic fibrosarcoma oncogene homolog*) may be involved in adipocyte differentiation and lipid metabolism regulation [36].

Including the *DGAT1* gene in the GWAS model confirmed important genomic regions associated with milk fatty acids. In addition, this approach highlighted the importance of accounting for major genes in GWAS, as the majority of significant effects were found for genes located in the BTA14, close to the *DGAT1* gene. Therefore, these results suggest that the *FOXH1* and *CYHR1* genes may be redundant signals of the *DGAT1* gene. These findings are in agreement with those of Capomaccio et al. [16], who also reported redundant signals of the *DGAT1* gene while studying milk production traits in three Italian cattle breeds.

Among the significant overlapping genomic regions found while fitting or not the *DGAT1* gene in the statistical model are the regions on BTA5, which are associated with the *MGST1* and *PLBD1* genes. In humans, the *MGST1* gene (*Microsomal glutathione transferase*) metabolizes hydrophobic substrates, such as phospholipid hydroperoxides and halogenated hydrocarbons [37], i.e., lipid and fatty acid hydroperoxides, lipid peroxidation products, and oxidized phospholipids are *MGST1* substrates [38]. In addition, rat liver microsomal glutathione transferase has the capacity to inhibit lipid peroxidation [39], and *MGST1* can detoxify several lipid peroxidation products as well as fatty acids [40]. However, the potential impact on milk lipid synthesis and/or secretion is still unknown. Previous studies have reported that the *PLBD1* gene (*Bos taurus* phospholipase B domain containing 1) is related to milk fat percentage in cattle [41]. Hayes et al. [33] also identified a region on BTA5 as significant while analyzing milk production traits including the SNP related to the *DGAT1* in the statistical model. Our findings indicate that *PLBD1* and *MGST1* may be important additional genes affecting milk fat composition in Holstein cattle. These candidate genes should be sequenced and further validated in independent populations. In addition to the genetic component influencing milk fatty acid composition, it is worth highlighting the fact that there are complementary alternatives to further alter milk fatty acid components, including through dietary changes [42,43,44].

## 5. Conclusions

Fitting the *DGAT1* gene effect as a covariate in the GWAS model confirmed some candidate genes previously associated with milk fatty acids in Holstein cattle, and allowed us to identify candidate genes in addition to the *DGAT1* gene, by removing redundant signals. The *PLBD1* and *MGST1* genes located on BTA5 are additional candidate genes affecting some groups of milk fatty acids, especially short- and medium-chain milk fatty acids. This study provides essential information on candidate genes and genomic regions affecting milk fatty acids in Holstein dairy cattle, and will serve as a basis for future studies on this regard.

## Figures and Tables

**Table 1:** Number of significant single nucleotide polymorphisms (chromosome-wide FDR = 1%, 5% and 10%) for the different groups of milk fatty acids, fitting or not the *DGAT1* gene in the statistical model.

Traits ^1^	Not Fitting *DGAT1* Gene Effect			Fitting *DGAT1* Gene Effect		
	1%	5%	10%	1%	5%	10%
LCFA	89	134	157	0	0	0
MCFA	204	252	338	0	0	3
SCFA	156	233	273	0	6	7
SFA	204	251	334	0	0	0
UFA	90	119	132	0	0	0

^1^ Groups of milk fatty acids: long-chain (LCFA), medium-chain (MCFA), short-chain (SCFA), saturated (SFA), and unsaturated (UFA) fatty acids.

## Data Availability

The data that support the findings of this study are presented in the paper and in the Supplementary file. The raw data cannot be made available, as it is property of the North American Holstein cattle producers and this information is commercially sensitive.

## Supplementary Materials

The following are available online at https://www.mdpi.com/2076-2615/9/11/997/s1, **Table S1**: Ranking of SNP variances when not fitting (model 1) or fitting (model 2) the *DGAT1* gene as a covariate in the genome-wide association analyses.
